# Transcriptome analysis of common and diverged circulating miRNAs between arterial and venous during aging

**DOI:** 10.18632/aging.103385

**Published:** 2020-06-30

**Authors:** Hao Wang, Yuan Zhou, Zhongnan Yin, Li Chen, Ling Jin, Qinghua Cui, Lixiang Xue

**Affiliations:** 1 Medical Research Center, Peking University Third Hospital, Beijing 100191, China; 2 Department of Biomedical Informatics, School of Basic Medical Sciences, Peking University, Beijing 100191, China; 3 Biobank, Peking University Third Hospital, Beijing 100191, China

**Keywords:** arterial-venous, circulating miRNA, aging, transcriptional factor

## Abstract

Circulating miRNAs have received extensive attention as non-invasive biomarkers for prediction and diagnosis of disease. However, most samples have been obtained from peripheral venous blood. To evaluate whether peripheral venous miRNAs represent circulating miRNAs from all blood vessels under a given condition, such as aging, we compared the miRNA profiles of venous and arterial plasma between young and aged rats by Illumina next-generation sequencing. The DEseq2 tool was used to obtain differentially-expressed miRNAs. We observed 105 aging-related deregulated miRNAs in vein and 62 in artery, which were highly associated with cell survival and inflammation, respectively. On the other hand, the young and aged groups exhibited a unique arterial-venous bias. There were 54 differentially-expressed miRNAs in the young group and 42 in the aged group; only 8 miRNAs were shared. Further transcriptional factors enrichment analysis found that the shared miRNAs could be partially upregulated by NF-κB and SIRT1. These transcriptional factors could be organ-specific and/or regulated in physiological and aging states as possible causal factors. This study suggested the potential application of circulating miRNAs, which reflect the systematic response to certain conditions, such as aging, and the importance of origin selection for candidate circulating miRNAs.

## INTRODUCTION

microRNA (miRNA), an endogenous, small, single-stranded, non-coding RNA molecule, plays important roles in multiple cellular behaviors and functions [1]. Cells release miRNAs into the peripheral blood as circulating miRNAs, which stably exist for long-distance communication [2]. Differential expression of miRNAs under aberrant conditions may reflect abnormal physiological and pathological changes and can be used as biomarkers [3].

Aging is an unavoidable physiological progression, leading to the decline of cellular functions, and is involved in many diseases, such as cardiovascular diseases, neurodegenerative diseases, and cancers [4, 5]. Accumulating evidence has demonstrated altered circulating miRNA profiles during the aging process [6–8]. The differential expression of these miRNAs may reflect the changes that occur in cells and tissues during aging [8], to promote or compensate for the aging process [9]. Thus, circulating miRNAs can be helpful in investigating the underlying mechanism of aging. As the degree of aging varies among different tissues within an individual [10], the profiles for a certain type of miRNA might also change depending on the location of blood collection.

Arterial and venous blood flows through different tissues and organs, which have specific miRNA expression patterns. Therefore, the circulating miRNA profiles of arteries and veins may have minor differences [11], which could be magnified in certain physiological and pathological conditions [12]. Most previous studies on circulating miRNAs have focused on miRNAs in the venous blood, owing to the accessibility and the large overlap between arteries and veins. Of note, the slight difference of miRNAs between arterial and venous blood could not be neglected under the precision medicine background.

In this study, we measured circulating miRNA profiles between young (8 weeks) and aged (22 months) male SD rats and between arterial and venous miRNAs by Illumina next-generation sequencing. A series of differentially expressed miRNAs in both arterial-versus-venous and aged-versus-young were identified. In addition, bioinformatic analysis provided an important clue for understanding the regulation and function of these differentially expressed miRNAs.

## RESULTS

### Functional and disease association analysis of deregulated circulating miRNAs in artery and vein during aging

**Figure 1:**
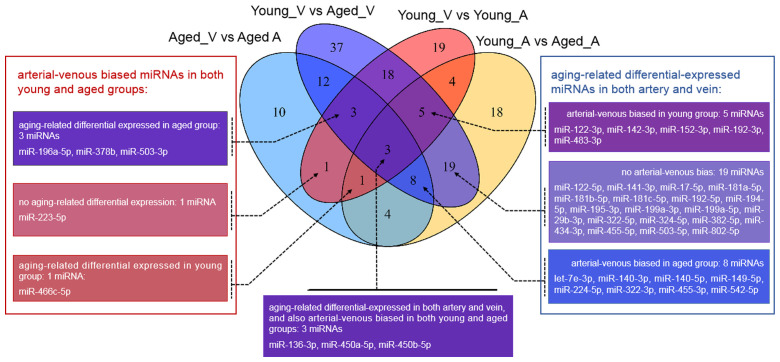
**Venn diagram showing the overlaps between deregulated miRNAs in different comparisons.** vs.: versus; A: arterial; V: venous.

**Figure 2:**
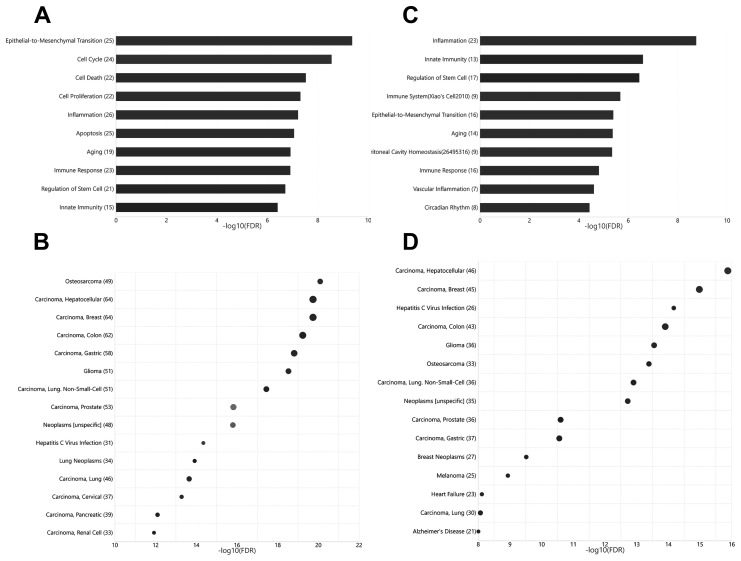
**Functional and disease associations of deregulated arterial and venous miRNAs between aged and young rats**. (**A**) Bar plot showing the functional association of deregulated venous miRNAs between aged and young rats. (**B**) Bubble plot showing the disease association of deregulated venous miRNAs between aged and young rats. (**C**) Bar plot showing the functional association of deregulated arterial miRNAs between aged and young rats. (**D**) Bubble plot showing the disease association of deregulated arterial miRNAs between aged and young rats. The number beside the function terms indicates the number of differentially expressed miRNAs associated with the corresponding function.

Current knowledge on circulating miRNAs is largely based on studies on venous blood samples. We first performed functional and disease association analysis of deregulated miRNAs between young and aged rats in venous blood. In total, 105 deregulated miRNAs (Figure 1), including 38 upregulated miRNAs and 67 downregulated miRNAs in aged rats, were observed (Supplementary Table 1). Functional enrichment analysis of deregulated miRNAs indicated a clear over-representation of known aging-related miRNAs (Figure 2A, FDR = 1.20E-7). In addition, functional terms related to epithelial-to-mesenchymal transition (EMT), cell proliferation (FDR = 4.33E-10), cell cycle (FDR = 2.81E-9), cell death (FDR = 2.98E-8), inflammation (FDR = 6.03E-8), and immune response (FDR = 1.22E-7) were also at the top of the list. We further analyzed the disease associations of the deregulated miRNAs. In line with the enriched functions of cell proliferation, cell death, and EMT, various types of cancers such as osteosarcoma (FDR = 8.18E-21), breast carcinoma (FDR = 1.84E-20), and hepatocellular carcinoma (FDR = 2.12E-20) dominated the list of associated diseases (Figure 2B). In addition, hepatitis C virus infection was the most significantly associated non-cancer disease term (FDR = 4.50E-15), which also agreed with the enriched function of the immune response.

Unlike that for circulating miRNAs in venous blood, current knowledge on circulating miRNAs in arterial blood is limited. Previously, we have reported similar, but not identical, expression patterns between circulating miRNAs in arterial and venous blood [11]. Indeed, deregulated miRNAs between young and aged rats in arterial blood prominently overlapped with those in venous blood (Figure 1, Supplementary Table 2): among 62 deregulated miRNAs (15 upregulated and 37 downregulated in aged rats), 35 were common with the venous blood results. Correspondingly, the functional enrichment of deregulated miRNAs from arterial blood was similar to that of deregulated miRNAs from venous blood (Figure 2C). For example, known aging-associated miRNAs were also enriched (FDR = 4.25E-6), and functional terms, such as epithelial-to-mesenchymal transition (FDR = 4.04E-6), inflammation (FDR = 1.77E-9), and immune response (FDR = 1.53E-5), ranked at the top. Similarly, shared disease associations, such as osteosarcoma (FDR = 4.10E-14), breast carcinoma (FDR = 1.05E-15), hepatocellular carcinoma (FDR = 1.33E-16), and hepatitis C virus infection (FDR = 6.74E-15), were also observed. Nevertheless, several functional and disease associations were uniquely represented in the top list for deregulated miRNAs in arterial blood (Figure 2C and 2D), including but not limited to functional terms, such as circadian rhythm (FDR = 3.69E-5) and vascular inflammation (FDR = 2.46E-5), and disease terms, such as heart failure (FDR = 7.69E-9) and Alzheimer’s disease (FDR = 9.88E-9). Indeed, significant differential expression between circulating miRNAs in arterial and venous blood was observed in both young and aged rats, which will be described in the following sections.

### Functional association analysis of differentially expressed miRNAs and their target genes in arterial and venous blood

**Figure 3:**
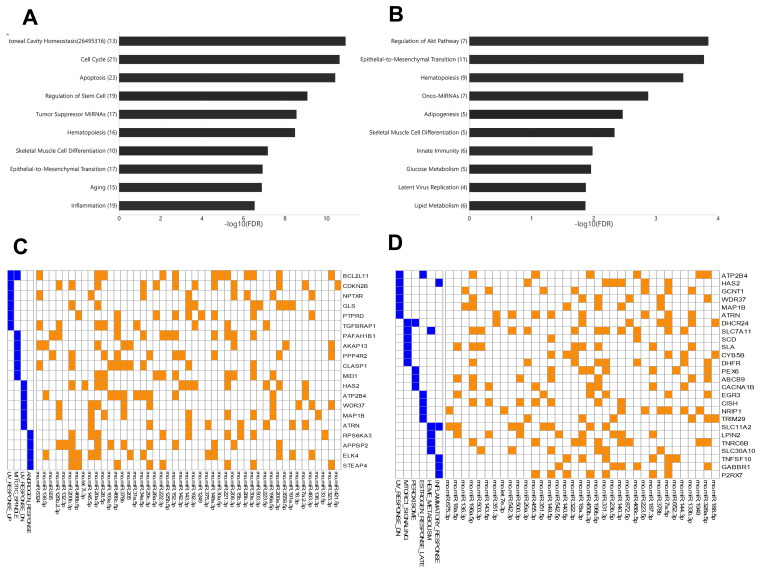
**The enriched functions of deregulated miRNAs between arterial and venous blood samples and their target genes.** (**A**) Bar plot showing the functional association of differentially-expressed miRNAs between arterial and venous plasma from young rats. (**B**) Bar plot showing the functional association of deregulated miRNAs between arterial and venous plasma from aged rats. (**C**) Heatmap showing the genes intensively regulated by deregulated genes in young rats and their functional association. The interaction between miRNAs and their target genes is indicated by an orange box, while the presence of the target gene in specific pathways is indicated by a blue box. (**D**) Heatmap showing the genes intensively regulated by deregulated genes in aged rats and their functional association. The number beside the function terms indicates the number of differentially expressed miRNAs associated with the corresponding function.

Among young rats, 54 miRNAs showed significant differential expression between arterial and venous blood samples, including 37 miRNAs that showed higher expression levels in arterial blood and 17 miRNAs that showed higher expression levels in venous blood (Supplementary Table 3). These miRNAs were enriched for functions (Figure 3A) such as peritoneal cavity homeostasis (FDR = 1.19E-11), cell cycle (FDR = 2.33 E-11), and apoptosis (FDR = 3.78E-11). Among aged rats, 42 miRNAs showed significant differential expression between arterial and venous blood samples, including 30 miRNAs that showed higher expression levels in arterial blood and 12 miRNAs that showed higher expression levels in venous blood (Supplementary Table 4). These miRNAs were enriched for functions (Figure 3B) such as regulation of the Akt pathway (FDR = 1.44E-4), epithelial-to-mesenchymal transition (FDR = 1.69E-4), and hematopoiesis (FDR = 3.63E-4). The Akt pathway is one of the well-known regulators of diabetes and cancer [13]. Indeed, these differentially expressed miRNAs showed significant associations with type 2 diabetes mellitus (FDR = 6.02E-5) and various types of cancers, such as gastric carcinoma (FDR = 1.03E-7) (data not shown).

To further identify genes specifically targeted by differentially expressed miRNAs, we analyzed the enriched functions of intensively regulated miRNA targets. The enriched functions of the target genes partly correlated with the enriched functions of differentially expressed miRNAs. For example, among intensively regulated miRNA targets in aged rats, genes involved in the mTOR pathway, a key downstream component of the Akt pathway [13], were significantly over-represented (HALLMARK_MTORC1_SIGNALING, FDR = 2.77E-2). Although there was no overlap between intensively regulated miRNA targets, one gene set was shared between intensively regulated miRNA targets found in young and aged rats (Figure 3C and 3D), that is, genes downregulated in response to ultraviolet irradiation (HALLMARK_UV_RESPONSE_DN, FDR = 3.63E-2 and 7.92E-3, respectively). Interestingly, we also observed divergent over-representation of sex steroid response pathways. Although intensively regulated targets in young rats were enriched for genes involved in response to androgen (HALLMARK_ANDROGEN_RESPONSE, FDR = 4.73E-2), those in aged rats were enriched for genes involved in late response to estrogen (HALLMARK_ESTROGEN_ RESPONSE_LATE, FDR = 4.38E-2).

### Consistent and reversed differential expression in arterial and venous blood between young and aged rats

**Figure 4:**
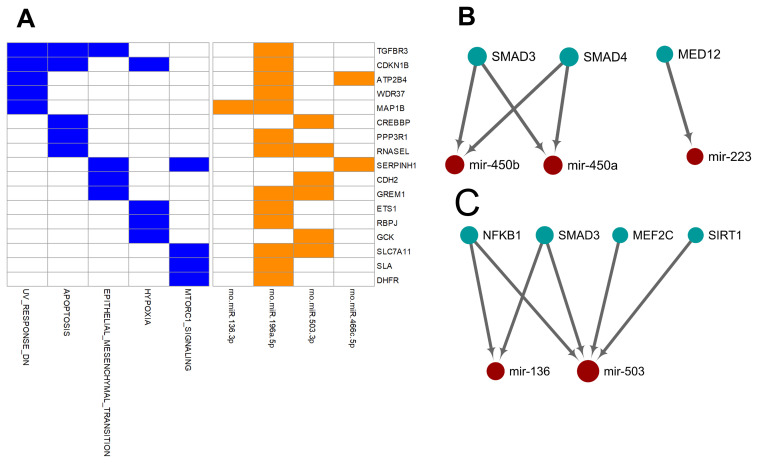
**Over-represented target genes and transcription factors of the miRNAs showed consistent or reversed artery-versus-venous deregulation between young and aged rats.** (**A**) Heatmap of genes intensively regulated by miRNAs showing reversed deregulation and their functional association. (**B**) Network view of the interactions between miRNAs showing consistent deregulation and their over-represented upstream transcription factors. (**C**) Network view of the interactions between miRNAs showing reversed deregulation and their over-represented upstream transcription factors.

Unlike the deregulated miRNAs observed in the aged-versus-young comparisons, differentially expressed miRNAs between arterial and venous blood samples showed limited overlaps between young and aged rats (Figure 1). Only eight differentially expressed miRNAs were shared. Three miRNAs, miR-450a-5p, miR-450b-5p, and miR-223-5p exhibited a consistent direction of differential expression. On the other hand, five miRNAs including miR-136-3p, miR-196a-5p, miR-466c-5p, miR-378b and miR-503-3p showed a reversed direction of differential expression. When analyzing the enriched functions among intensively regulated genes, the consistent miRNAs tended to target pancreatic beta cell genes (HALLMARK_PANCREAS_BETA_CELLS, FDR = 2.16E-2). On the other hand, the reverse miRNAs (Figure 4A) tended to target genes downregulated in response to ultraviolet irradiation (HALLMARK_UV_RESPONSE_DN, FDR = 1.78E-3), apoptosis (HALLMARK_APOPTOSIS, FDR = 1.78E-3), epithelial-to-mesenchymal transition (HALL MARK_EPITHELIAL_MESENCHYMAL_TRANSITION, FDR = 1.99E-2), hypoxia response (HALL MARK_HYPOXIA, FDR = 1.99E-2), and mTOR signaling (HALLMARK_MTORC1_SIGNALING, FDR = 1.99E-2).

Finally, we try to explore the plausible causal factors of consistent and reversed miRNA differential expression between arterial and venous blood by analyzing the enriched upstream transcription factors. The results have been illustrated in Figure 4B and 4C, respectively. From these regulatory networks, consistent differential expression of miR-450a-5p, miR-450b-5p, and miR-223-5p was found to be partially under the regulation of SMAD3, SMAD4, and MED-12 (Figure 4B), whereas reversed differential expression of miR-136-3p and miR-503-3p was partially regulated by NF-κB1, SMAD3, MEF2C, and SIRT1 (Figure 4C).

### Validation of deregulated miRNAs

**Figure 5:**
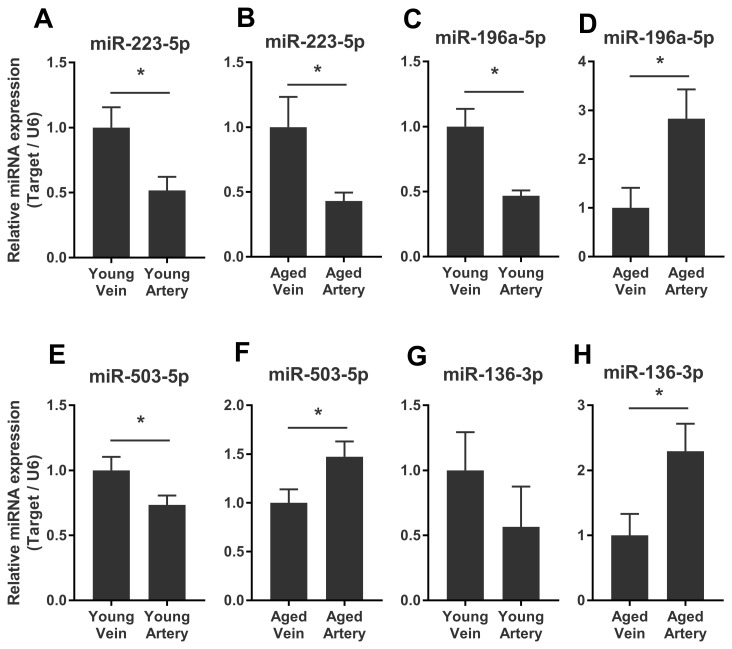
**Validation of consistent and reversed arterial-versus-venous deregulated miRNAs between young and aged rats.** (**A**–**B**) levels of miR-223-5p in venous and arterial plasma of young (**A**) and aged (**B**) rats. (**C**–**D**) levels of miR-196a-5p in venous and arterial plasma of young (**C**) and aged (**D**) rats. (**E**–**F**) levels of miR-503-3p in venous and arterial plasma of young (**E**) and aged (**F**) rats. (**G**–**H**) levels of miR-136-3p in the venous and arterial plasma of young (**G**) and aged (**H**) rats. Data are represented as mean ± SEM. *: p<0.05.

We validated some of the overlapped miRNAs with arterial or venous bias between young and aged rats. We were interested in shared arterial-venous biased miRNAs between the young and aged groups. The expression patterns of the eight miRNAs described above were measured. In consistent with the sequencing results, miR-223-5p displayed a similar arterial-versus-venous trend in both young and aged rats. The level of this small RNA molecule was lower in the artery than in the vein (Figure 5A, 5B). For the reversed arterial-venous bias miRNAs, expressions of miR-196a-5p and miR-503-3p were validated. In the young group, these two miRNAs showed reduced levels in the artery (Figure 5C, 5E). However, in the aged group, these tendencies were reversed (Figure 5D, 5F). miR-136-3p in the young group also showed lower level in the artery (Figure 5G). In the aged group, this miRNA showed elevated tendency without significant difference (Figure 5H). Thus, the origin of circulating miRNAs may influence their expression pattern, which could be important when discussing the functional mechanism and clinical biomarker potential of miRNAs of interest.

## DISCUSSION

### Aging-related miRNAs are highly associated with the immune response and cancer in both artery and vein

Aging is a universal process that involves progressive tissue and organ dysfunction [4, 5]. This complex biological phenomenon correlates with various molecular changes, such as genomic instability and epigenetic remodeling, which in turn effect cellular dysregulation, including metabolic stress, oxidative stress, autophagy, unfolded protein response, chronic low-grade inflammation, and cellular senescence [4, 5, 14]. Many diseases have a higher correlation with aging, including vascular dysfunction, autoimmune diseases, neurodegenerative diseases, type 2 diabetes mellitus, obesity, and cancer [5, 14, 15]. Large numbers of factors exhibit altered expression patterns under aging conditions. One of them is miRNA, a non-coding small RNA molecule that is produced by almost all cell types. The expression levels of circulating miRNAs are associated with age and age-related diseases [16, 17], and some of them have been identified as candidate biomarkers of age-associated diseases for diagnosis and prognosis [18, 19].

Functional enrichment analysis revealed that deregulated miRNAs derived from vein and artery correlated with aging-related miRNAs, EMT, stem cell regulation, and terms related to immunity (inflammation, innate immunity, and immune response) (Figure 2A, 2C). All these cell behaviors are closely related to the aging process. Multiple studies have focused on the complex interactions between aging, the immune system, and inflammation. With the progression of aging, the immune system undergoes a gradual decline in functional capacity (called immunosenescence) and chronic low-grade inflammation without exogenous infection (called inflammaging) [20–22]. Aging leads to defects in the phenotypes, function, development, and maturation of immune cells [20]. On the other hand, senescent cells release a series of pro-inflammatory factors such as senescence-associated secretory phenotype factors [23], including miRNAs. Many inflammation-related miRNAs have also been implicated in aging, like miR-21 and miR-126a [24, 25]. In addition, elevated levels of miR-34b were observed in aging macrophages, which exhibited an impaired immune response [26].

Disease enrichment analysis showed that aging-related deregulated miRNAs in vein and artery highly correlated with hepatitis C virus infection and various types of cancer, such as osteosarcoma, breast carcinoma, and hepatocellular carcinoma (shown in Figure 2B and 2D). This coincided with the association between aging and immunity mentioned above. The deficient immune response of the aged impairs anticancer immunity and increases the prevalence of cancers. Increasing age is also associated with higher susceptibility to genomic mutations harbored by senescent cells during their lifetimes. With the accumulated oncogenic mutations, senescent cells change their characteristics, such as cytoskeletal structures, expression of lysosomal enzymes, and metabolic patterns, and escape apoptosis to become tumor cells. Senescence of human adipose tissue stem cells was shown to lead to the upregulation of the tumor suppressive miR-100 cluster and the oncogenic miR-17~92 cluster [9]. Their aberrant expression conversely modulated the senescence phenotype by regulating cell cycle, chromatin, transcription and translation, and histone methyltransferases. Interestingly, our data showed that four members of the miR-17~92 cluster were differentially expressed in the venous plasma of aged rats. However, for arterial plasma, only miR-17-5p changed in the aged group.

### Venous and arterial aging-related deregulated miRNAs are associated with cell survival, and vascular inflammation, respectively

Highly similar but slightly diverse miRNA profiles have been reported to exist in different vascular locations. For example, twenty-four differentially-expressed miRNAs were previously reported between arterial and venous plasma of ten-week-old male SD rats [11]. Endothelial cells from distinct locations exhibit baseline miRNA diversity [27], which could contribute to the arterial-venous bias. In addition, the baseline level of miRNAs in blood may be influenced by the path of blood flow, as different tissues and organs have specific expression patterns. Moreover, certain organs may also retain miRNAs present in the blood flowing in. Let-7g, miR-15b, miR-155, and miR-328 are present at higher levels in mesenteric than peripheral vein plasma in colon cancer patients, implying that they are likely retained in the liver [28]. Aberrant or pathologic conditions further affect miRNA expression patterns in different tissues. miR-140 has been shown to be differentially expressed in arterial and coronary sinus blood in healthy people. However, for patients with heart failure, 15 differentially expressed miRNAs were detected, implying altered miRNA secretion from the heart into the coronary sinus [12]. Another study suggested that the extracellular vesicles in arterial and venous sera of patients with heart disease showed highly similar miRNA expression profiles [29]. As circulating miRNAs can be stably carried by both vesicles and protein complexes [2], and the serum was more abundant in platelet-derived miRNAs than the corresponding plasma [30], the contradictory results observed could be due to sample types. Therefore, blood miRNA profiles in certain locations may be a concerto of the flow path, the vessel status, and physiology or pathology status and influenced by the type of blood sampling.

Aging is a systematic progressive deterioration. Owing to the different responses and cellular behaviors during the aging process, alterations in miRNA expression and secretion could be distinct in different types of cells, tissues, and organs [10]. Thus, changes in miRNA profiles may be quite complicated. In our study, several functional terms were uniquely presented for arterial or venous aging-associated miRNAs. The top-ranking artery-specific functional term was vascular inflammation, which could be due to vascular senescence. Compared with veins, arterial vessels show more atherosclerotic plaques and more endothelial cell dysfunction during the aging process [31, 32], suggesting that differential expression of vascular inflammation-related miRNAs was probably easier to observe in arteries. On the other hand, the functional terms uniquely presented in the top list of venous deregulated miRNAs were cell cycle, cell proliferation, cell death, and cell apoptosis. We speculated that these differentially expressions were more likely to reflect changes downstream of the flow path.

### The functions of aging-related miRNAs differed between arterial- and venous-origin

In this study, we also observed differential miRNA expression between arterial and venous plasma in both young (8-weeks-old) and aged (22-months-old) male rats (Figure 1). Among all arterial-venous differentially-expressed miRNAs, six miRNAs overlapped with those reported previously [11]. Figure 3 shows the enrichment analysis of arterial-venous biased miRNAs in young rats (Figure 3A) and aged rats (Figure 3B). The enriched functional terms, hematopoiesis and skeletal muscle differentiation, were shared by both groups, indicating that lifespan may be less correlated than vascular location for these terms, or their change during aging may be universal. Unique, top ranked functions in the aged group included regulation of metabolism, such as glucose metabolism, lipid metabolism, and adipogenesis, whereas in the young group, these were cellular activities, such as cell cycle, apoptosis, and stem cell regulation. Arterial-venous biased miRNAs were also enriched for onco-miRNAs and native immune cells in the aged group and for tumor suppressor miRNA and inflammation in the young group. The Akt pathway was specifically associated with arterial-venous biased miRNAs in aged rats instead of the young ones, implying differential modulation between arterial- and venous-circulating miRNA profiles during the aging process. These observations suggested that different organs or tissues can undergo aging differently, and deregulated miRNAs detected in blood could be referred to different vascular locations.

Interestingly, although many associated functional terms were shared or related in young and aged groups, each arterial-venous differentially expressed miRNA profile was unique, and only eight miRNAs overlapped (Figure 1). miR-223-5p, miR-450a-5p, and miR-450b-5p showed consistent tendencies in the two groups. These three miRNAs were associated with pancreatic β cells, a term beyond the top-ranking list of aging-related miRNAs or arterial-venous biased miRNAs. Another interesting observation was that five miRNAs (miR-136-3p, miR-196a-5p, miR-378b, miR-466c-5p, and miR-503-3p) showed reversed arterial-venous bias between the young and aged groups. They were highly associated with apoptosis, EMT, hypoxia, and the mTOR pathway. These cellular behaviors and response pathways were influenced by the aging process. EMT has been shown to decrease in senescent cells [33], whereas mTOR [34, 35] and hypoxia-related pathways [36, 37] have been shown to be induced. Apoptosis and senescence are cellular responses to damage with different mechanisms; both are involved in aging, and their relationship may vary under different conditions [38, 39]. It is noteworthy that these reversed miRNAs made up only a small proportion of differentially-expressed miRNAs (5/54 for young and 5/42 for aged), implying limited impact on total functions and regulation. However, the reversed bias we observed may imply a diverse degree of response to aging in different cellular and vascular environments.

### The deregulation of miRNAs probably resulted from upstream transcriptional factors

Furthermore, we tried to identify the transcriptional factors that led to the differentially-expressed miRNA profiles. We found that the two strands of a few aging-related miRNAs changed synchronously, accounting for 23% (14/62) and 32% (34/105) miRNAs in the arterial and venous plasma, respectively. For arterial-venous biased miRNAs, miR-29c-5p and -3p were both arterial-biased in the young group, whereas five pairs of miRNAs (miR-18a-5p/3p, miR-351-5p/3p, miR-542-5p/3p, miR-450b-5p/3p, and miR-140-5p/3p) were higher in the arterial plasma of the aged group. The synchronous deregulation of the two strands could be consistent with the function of transcriptional factors that regulate the transcription of primary miRNA transcripts. For miRNAs with only one strand deregulated, altered expression levels could also reflect the spatial and temporal differentiation of their upstream transcriptional factors during aging and in various vascular locations. Among all differentially expressed miRNAs, we focused on the eight overlapping arterial-versus-venous differentially-expressed miRNAs mentioned above as they showed vascular origin-bias in both young and aged rats. The TransmiR v2.0 tool inferred that three consistent miRNAs were under the control of SMAD3, SMAD4, and MED12, whereas the reverse was regulated by SMAD3, MEF2C, NF-κB, and SIRT1 (shown in Figure 4). These factors dysregulated during the aging process, which was bound to further affect the downstream miRNA they modulated. SIRT1 [40–42] and MEF2C [43] decrease with age. Translocation of NF-κB into the nucleus is augmented in old SAMP8 mice [44], and elevation of NF-κB activity regulates pro-inflammatory response in aged mice and the human artery by releasing TNF-α, IL-1β, IL-6, and IFN-γ [45]. For predicted transcriptional factors of consistent miRNAs, we speculated that their dysfunction in the aging process might be similar across various types of tissues and did not significantly alter the basal difference between arteries and veins. However, for reversed miRNAs and their enriched upstream transcriptional factors, effects of aging on their expression or activity or both might be large enough to conceal or override the influence of the origin of the vessel. It could cause drastic changes in one or several tissues, or from the combination of multiples.

Transcriptional factors are mainly responsible for regulating the expression of downstream encoding proteins. In many cases, localized dysfunctions are difficult to detect and circulating biomarkers of appropriate origin give us a chance to evaluate the given location *in situ*. Further investigation is needed to deepen our understanding of the influence of aging under different conditions from the transcription point of view.

In brief, considering their accessibility, venous miRNAs are ideal for liquid biopsy-based analysis. Several studies have identified miRNA candidates as biomarkers for different diseases. However, our present work indicates that subtle differences between venous and arterial miRNAs were enlarged during aging. This fact reminds us of the necessity of a more precise comparison of miRNAs from different origins and under different scenarios. Therefore, re-evaluation of the efficiency of candidate miRNAs as biomarkers should be based on differences in disease states or origin of disease. Overall circumstances should be evaluated before using certain miRNAs as biomarkers.

## MATERIALS AND METHODS

### Animal protocols and ethics statement

Male SD rats were obtained from Tianqin Biotech Co (Changsha, China). Animal housing and handling were performed in accordance with good laboratory practice in Animal Department, Health Science Center, Peking University. Briefly, All of the rats were housed in standard cases in the environment where temperature- and humidity-controlled and on a 12 hrs light/dark cycle before initiating the experiments. The rats aged 8 weeks and 22 months were used as young group and aged group respectively. Animal care and experimental procedures were done with the approval of Peking University Animal Ethics Committee.

### miRNA transcriptome profiling

Rats were anaesthetized using pentobarbital sodium; arterial and venous blood was obtained from the abdominal aorta and abdominal main vein, respectively. The sample size was five samples per group (aged arterial, aged venous, young arterial, young venous, and 20 samples in total). To collect arterial and venous plasma, EDTA-anticoagulant blood was centrifuged at 2,000 *g* for 15 min at 4 °C, and the supernatant was stored at -80 °C. Total RNA was isolated using the TRIzol reagent (Invitrogen) according to the manufacturer’s instructions.

RNA samples were prepared using the TruSeq Small RNA Sample Prep Kit (Illumina) and the miRNA transcriptomes were profiled using small RNA sequencing techniques provided by Majorbio, Shanghai, China. The clean reads, obtained after quality control and removal of adaptor sequences, were aligned to the rat genome and the expression levels of known miRNAs from miRBase (http://www.mirbase.org, release 21) were quantified and normalized in TPM, as described previously [46]. A total of 496 known miRNAs from miRbase were detected by small RNA-seq and used as the input for differential expression analysis. Finally, differentially expressed miRNAs were obtained using the DEseq2 tool, which is based on Empirical Bayes shrinkage for fold change estimation and Wald test for statistical significance evaluation [47], with the recommended parameters. At least two-fold change, in combination with P-value < 0.05, was applied as the threshold for differentially-expressed miRNAs, whereas the corrected P-values via Benjamini-Hochberg false discovery rate (FDR, calculated by using ‘p.adjust’ function in R) were presented as a reference [48] (Supplementary Tables 1–4). The datasets generated and analyzed in the current study are available in the GEO repository (GSE138317).

### Functional enrichment analysis of differentially expressed miRNAs and their target genes

We used the TAM 2.0 server [49] to analyze significant disease associations of deregulated miRNAs. We also investigated the enriched functions of the target genes of deregulated miRNAs. First, potential target genes of differentially expressed miRNAs were predicted using miRanda [50], and then genes were sorted by the number of targeting deregulated miRNAs. Thereafter, the top 15% of intensively regulated genes targeted by deregulated miRNAs were selected for further functional enrichment analysis. Functional enrichment analysis was performed by comparing with the MSigDB Hallmark Gene Set reference [51] using default parameters. The results were visualized as heatmaps with the ‘pheatmap’ package in R. Finally, we analyzed enriched upstream transcription factors (TFs) among the deregulated miRNAs by using TransmiR v2.0 tool [52] with the limitation of considering high confidence transcriptional factor-miRNA regulation sets (‘set level 2’) only, and the regulatory network between transcriptional factors and miRNAs were visualized by Cytoscape [53]. The Benjamini-Hochberg FDR method was applied for multiple correction adjustment by using the ‘p.adjust’ function in R.

### Validation of differentially expressed miRNAs

Validation of deregulated miRNAs was performed by quantitative real-time PCR (qRT-PCR). Ten aged rats and five young rats were newly introduced. Total RNA was reverse-transcribed to cDNA using the MiRcute miRNA First-strand cDNA synthesis kit (Tiangen Biotech Co). qRT-PCR was performed using MiRcute MiRNA Premix (Tiangen Biotech Co). The statistical analyses were performed with SPSS 17.0 software. Comparisons among groups were evaluated by Student’s t-test. P < 0.05 was considered as significant difference.

## Supplementary Material

Supplementary Table 1

Supplementary Tables 2-4

## References

[1] Bushati N, Cohen SM. microRNA functions. Annu Rev Cell Dev Biol. 2007; 23:175–205. 10.1146/annurev.cellbio.23.090506.123406 17506695

[2] Arroyo JD, Chevillet JR, Kroh EM, Ruf IK, Pritchard CC, Gibson DF, Mitchell PS, Bennett CF, Pogosova-Agadjanyan EL, Stirewalt DL, Tait JF, Tewari M. Argonaute2 complexes carry a population of circulating microRNAs independent of vesicles in human plasma. Proc Natl Acad Sci USA. 2011; 108:5003–8. 10.1073/pnas.1019055108 21383194 PMC3064324

[3] Wang H, Peng R, Wang J, Qin Z, Xue L. Circulating microRNAs as potential cancer biomarkers: the advantage and disadvantage. Clin Epigenetics. 2018; 10:59. 10.1186/s13148-018-0492-1 29713393 PMC5913875

[4] Barroso-Vilares M, Logarinho E. Chromosomal instability and pro-inflammatory response in aging. Mech Ageing Dev. 2019; 182:111118. 10.1016/j.mad.2019.111118 31102604

[5] Levy D, Reichert CO, Bydlowski SP. Paraoxonases activities and polymorphisms in elderly and old-age diseases: an overview. Antioxidants (Basel). 2019; 8:118. 10.3390/antiox8050118 31052559 PMC6562914

[6] Ameling S, Kacprowski T, Chilukoti RK, Malsch C, Liebscher V, Suhre K, Pietzner M, Friedrich N, Homuth G, Hammer E, Völker U. Associations of circulating plasma microRNAs with age, body mass index and sex in a population-based study. BMC Med Genomics. 2015; 8:61. 10.1186/s12920-015-0136-7 26462558 PMC4604724

[7] Garza-Manero S, Arias C, Bermúdez-Rattoni F, Vaca L, Zepeda A. Identification of age- and disease-related alterations in circulating miRNAs in a mouse model of alzheimer’s disease. Front Cell Neurosci. 2015; 9:53. 10.3389/fncel.2015.00053 25745387 PMC4333818

[8] Olivieri F, Capri M, Bonafè M, Morsiani C, Jung HJ, Spazzafumo L, Viña J, Suh Y. Circulating miRNAs and miRNA shuttles as biomarkers: perspective trajectories of healthy and unhealthy aging. Mech Ageing Dev. 2017; 165:162–70. 10.1016/j.mad.2016.12.004 27986629 PMC5481482

[9] Lopez MF, Niu P, Wang L, Vogelsang M, Gaur M, Krastins B, Zhao Y, Smagul A, Nussupbekova A, Akanov AA, Jordan IK, Lunyak VV. Opposing activities of oncogenic MIR17HG and tumor suppressive MIR100HG clusters and their gene targets regulate replicative senescence in human adult stem cells. NPJ Aging Mech Dis. 2017; 3:7. 10.1038/s41514-017-0006-y 28649425 PMC5460214

[10] Stegeman R, Weake VM. Transcriptional signatures of aging. J Mol Biol. 2017; 429:2427–37. 10.1016/j.jmb.2017.06.019 28684248 PMC5662117

[11] Xu W, Zhou Y, Xu G, Geng B, Cui Q. Transcriptome analysis reveals non-identical microRNA profiles between arterial and venous plasma. Oncotarget. 2017; 8:28471–80. 10.18632/oncotarget.15310 28212530 PMC5438665

[12] Marques FZ, Vizi D, Khammy O, Mariani JA, Kaye DM. The transcardiac gradient of cardio-microRNAs in the failing heart. Eur J Heart Fail. 2016; 18:1000–8. 10.1002/ejhf.517 27072074

[13] Zoncu R, Efeyan A, Sabatini DM. mTOR: from growth signal integration to cancer, diabetes and ageing. Nat Rev Mol Cell Biol. 2011; 12:21–35. 10.1038/nrm3025 21157483 PMC3390257

[14] de Pablos RM, Espinosa-Oliva AM, Hornedo-Ortega R, Cano M, Arguelles S. Hydroxytyrosol protects from aging process via AMPK and autophagy; a review of its effects on cancer, metabolic syndrome, osteoporosis, immune-mediated and neurodegenerative diseases. Pharmacol Res. 2019; 143:58–72. 10.1016/j.phrs.2019.03.005 30853597

[15] Uddin MS, Kabir MT, Jakaria M, Mamun AA, Niaz K, Amran MS, Barreto GE, Ashraf GM. Correction to: endothelial PPARγ is crucial for averting age-related vascular dysfunction by stalling oxidative stress and ROCK. Neurotox Res. 2019; 36:437–38. 10.1007/s12640-019-00065-3 31134581

[16] Rani A, O'Shea A, Ianov L, Cohen RA, Woods AJ, Foster TC. miRNA in circulating microvesicles as biomarkers for age-related cognitive decline. Front Aging Neurosci. 2017; 9:323. 10.3389/fnagi.2017.00323 29046635 PMC5632661

[17] Olivieri F, Albertini MC, Orciani M, Ceka A, Cricca M, Procopio AD, Bonafè M. DNA damage response (DDR) and senescence: shuttled inflamma-miRNAs on the stage of inflamm-aging. Oncotarget. 2015; 6:35509–21. 10.18632/oncotarget.5899 26431329 PMC4742121

[18] Cardoso AL, Fernandes A, Aguilar-Pimentel JA, de Angelis MH, Guedes JR, Brito MA, Ortolano S, Pani G, Athanasopoulou S, Gonos ES, Schosserer M, Grillari J, Peterson P, et al. Towards frailty biomarkers: candidates from genes and pathways regulated in aging and age-related diseases. Ageing Res Rev. 2018; 47:214–77. 10.1016/j.arr.2018.07.004 30071357

[19] Kumar S, Vijayan M, Bhatti JS, Reddy PH. MicroRNAs as peripheral biomarkers in aging and age-related diseases. Prog Mol Biol Transl Sci. 2017; 146:47–94. 10.1016/bs.pmbts.2016.12.013 28253991

[20] Salminen A, Kaarniranta K, Kauppinen A. Immunosenescence: the potential role of myeloid-derived suppressor cells (MDSC) in age-related immune deficiency. Cell Mol Life Sci. 2019; 76:1901–18. 10.1007/s00018-019-03048-x 30788516 PMC6478639

[21] Flynn MG, Markofski MM, Carrillo AE. Elevated inflammatory status and increased risk of chronic disease in chronological aging: inflamm-aging or Inflamm-inactivity? Aging Dis. 2019; 10:147–56. 10.14336/AD.2018.0326 30705775 PMC6345337

[22] Mancuso P, Bouchard B. The impact of aging on adipose function and adipokine synthesis. Front Endocrinol (Lausanne). 2019; 10:137. 10.3389/fendo.2019.00137 30915034 PMC6421296

[23] Prata LG, Ovsyannikova IG, Tchkonia T, Kirkland JL. Senescent cell clearance by the immune system: emerging therapeutic opportunities. Semin Immunol. 2018; 40:101275. 10.1016/j.smim.2019.04.003 31088710 PMC7061456

[24] Kim C, Hu B, Jadhav RR, Jin J, Zhang H, Cavanagh MM, Akondy RS, Ahmed R, Weyand CM, Goronzy JJ. Activation of miR-21-regulated pathways in immune aging selects against signatures characteristic of memory T cells. Cell Rep. 2018; 25:2148–62.e5. 10.1016/j.celrep.2018.10.074 30463012 PMC6371971

[25] Yan Y, Qin D, Hu B, Zhang C, Liu S, Wu D, Huang W, Huang X, Wang L, Chen X, Zhang L. Deletion of miR-126a promotes hepatic aging and inflammation in a mouse model of cholestasis. Mol Ther Nucleic Acids. 2019; 16:494–504. 10.1016/j.omtn.2019.04.002 31051334 PMC6495079

[26] Liang W, Gao S, Liang L, Huang X, Hu N, Lu X, Zhao Y. miRNA-34b is directly involved in the aging of macrophages. Aging Clin Exp Res. 2017; 29:599–607. 10.1007/s40520-016-0611-9 27538833

[27] McCall MN, Kent OA, Yu J, Fox-Talbot K, Zaiman AL, Halushka MK. MicroRNA profiling of diverse endothelial cell types. BMC Med Genomics. 2011; 4:78. 10.1186/1755-8794-4-78 22047531 PMC3223144

[28] Monzo M, Santasusagna S, Moreno I, Martinez F, Hernández R, Muñoz C, Castellano JJ, Moreno J, Navarro A. Exosomal microRNAs isolated from plasma of mesenteric veins linked to liver metastases in resected patients with colon cancer. Oncotarget. 2017; 8:30859–69. 10.18632/oncotarget.16103 28415718 PMC5458173

[29] Hermann S, Buschmann D, Kirchner B, Borrmann M, Brandes F, Kotschote S, Bonin M, Lindemann A, Reithmair M, Schelling G, Pfaffl MW. Transcriptomic profiling of cell-free and vesicular microRNAs from matched arterial and venous sera. J Extracell Vesicles. 2019; 8:1670935. 10.1080/20013078.2019.1670935 31632620 PMC6781181

[30] Wang K, Yuan Y, Cho JH, McClarty S, Baxter D, Galas DJ. Comparing the MicroRNA spectrum between serum and plasma. PLoS One. 2012; 7:e41561. 10.1371/journal.pone.0041561 22859996 PMC3409228

[31] Corada M, Morini MF, Dejana E. Signaling pathways in the specification of arteries and veins. Arterioscler Thromb Vasc Biol. 2014; 34:2372–77. 10.1161/ATVBAHA.114.303218 25169934

[32] dela Paz NG, D’Amore PA. Arterial versus venous endothelial cells. Cell Tissue Res. 2009; 335:5–16. 10.1007/s00441-008-0706-5 18972135 PMC4105978

[33] Wang Y, Fu B, Sun X, Li D, Huang Q, Zhao W, Chen X. Differentially expressed microRNAs in bone marrow mesenchymal stem cell-derived microvesicles in young and older rats and their effect on tumor growth factor-β1-mediated epithelial-mesenchymal transition in HK2 cells. Stem Cell Res Ther. 2015; 6:185. 10.1186/s13287-015-0179-x 26415502 PMC4587922

[34] Sahin K, Orhan C, Tuzcu M, Tastan H, Bilir B, Sahin N, Oner DA, Kucuk O. Tomato powder modulates NF- κ B, mTOR, and Nrf2 pathways during aging in healthy rats. J Aging Res. 2019; 2019:1643243. 10.1155/2019/1643243 30719353 PMC6334329

[35] Zhan JK, Wang YJ, Li S, Wang Y, Tan P, He JY, Chen YY, Deng HQ, Huang W, Lin X, Liu YS. AMPK/TSC2/mTOR pathway regulates replicative senescence of human vascular smooth muscle cells. Exp Ther Med. 2018; 16:4853–58. 10.3892/etm.2018.6767 30542441 PMC6257130

[36] Ebersole JL, Novak MJ, Orraca L, Martinez-Gonzalez J, Kirakodu S, Chen KC, Stromberg A, Gonzalez OA. Hypoxia-inducible transcription factors, HIF1A and HIF2A, increase in aging mucosal tissues. Immunology. 2018; 154:452–64. 10.1111/imm.12894 29338076 PMC6002220

[37] Ryu DR, Yu MR, Kong KH, Kim H, Kwon SH, Jeon JS, Han DC, Noh H. Sirt1-hypoxia-inducible factor-1α interaction is a key mediator of tubulointerstitial damage in the aged kidney. Aging Cell. 2019; 18:e12904. 10.1111/acel.12904 30614190 PMC6413666

[38] Childs BG, Baker DJ, Kirkland JL, Campisi J, van Deursen JM. Senescence and apoptosis: dueling or complementary cell fates? EMBO Rep. 2014; 15:1139–53. 10.15252/embr.201439245 25312810 PMC4253488

[39] Aravinthan AD, Alexander GJ. Senescence in chronic liver disease: is the future in aging? J Hepatol. 2016; 65:825–34. 10.1016/j.jhep.2016.05.030 27245432

[40] Jia G, Aroor AR, Jia C, Sowers JR. Endothelial cell senescence in aging-related vascular dysfunction. Biochim Biophys Acta Mol Basis Dis. 2019; 1865:1802–09. 10.1016/j.bbadis.2018.08.008 31109450

[41] Chuang PY, Cai W, Li X, Fang L, Xu J, Yacoub R, He JC, Lee K. Reduction in podocyte SIRT1 accelerates kidney injury in aging mice. Am J Physiol Renal Physiol. 2017; 313:F621–28. 10.1152/ajprenal.00255.2017 28615249 PMC5625108

[42] Yan J, Luo A, Gao J, Tang X, Zhao Y, Zhou B, Zhou Z, Li S. The role of SIRT1 in neuroinflammation and cognitive dysfunction in aged rats after anesthesia and surgery. Am J Transl Res. 2019; 11:1555–68. 30972182 PMC6456566

[43] Deczkowska A, Matcovitch-Natan O, Tsitsou-Kampeli A, Ben-Hamo S, Dvir-Szternfeld R, Spinrad A, Singer O, David E, Winter DR, Smith LK, Kertser A, Baruch K, Rosenzweig N, et al. Mef2C restrains microglial inflammatory response and is lost in brain ageing in an IFN-I-dependent manner. Nat Commun. 2017; 8:717. 10.1038/s41467-017-00769-0 28959042 PMC5620041

[44] Forman K, Vara E, García C, Kireev R, Cuesta S, Acuña-Castroviejo D, Tresguerres JA. Influence of aging and growth hormone on different members of the NFkB family and IkB expression in the heart from a murine model of senescence-accelerated aging. Exp Gerontol. 2016; 73:114–20. 10.1016/j.exger.2015.11.005 26581911

[45] Donato AJ, Morgan RG, Walker AE, Lesniewski LA. Cellular and molecular biology of aging endothelial cells. J Mol Cell Cardiol. 2015; 89:122–35. 10.1016/j.yjmcc.2015.01.021 25655936 PMC4522407

[46] Zhou L, Chen J, Li Z, Li X, Hu X, Huang Y, Zhao X, Liang C, Wang Y, Sun L, Shi M, Xu X, Shen F, et al. Integrated profiling of microRNAs and mRNAs: microRNAs located on Xq27.3 associate with clear cell renal cell carcinoma. PLoS One. 2010; 5:e15224. 10.1371/journal.pone.0015224 21253009 PMC3013074

[47] Wang L, Feng Z, Wang X, Wang X, Zhang X. DEGseq: an R package for identifying differentially expressed genes from RNA-seq data. Bioinformatics. 2010; 26:136–8. 10.1093/bioinformatics/btp612 19855105

[48] Benjamini Y, Hochberg Y. Controlling the false discovery rate: a practical and powerful approach to multiple testing. J R Stat Soc Ser B. 1995; 57:289–300. 10.1111/j.2517-6161.1995.tb02031.x

[49] Li J, Han X, Wan Y, Zhang S, Zhao Y, Fan R, Cui Q, Zhou Y. TAM 2.0: tool for MicroRNA set analysis. Nucleic Acids Res. 2018; 46:W180–85. 10.1093/nar/gky509 29878154 PMC6031048

[50] Betel D, Wilson M, Gabow A, Marks DS, Sander C. The microRNA.org resource: targets and expression. Nucleic Acids Res. 2008; 36:D149–53. 10.1093/nar/gkm995 18158296 PMC2238905

[51] Liberzon A, Birger C, Thorvaldsdóttir H, Ghandi M, Mesirov JP, Tamayo P. The molecular signatures database (MSigDB) hallmark gene set collection. Cell Syst. 2015; 1:417–25. 10.1016/j.cels.2015.12.004 26771021 PMC4707969

[52] Tong Z, Cui Q, Wang J, Zhou Y. TransmiR v2.0: an updated transcription factor-microRNA regulation database. Nucleic Acids Res. 2019; 47:D253–58. 10.1093/nar/gky1023 30371815 PMC6323981

[53] Su G, Morris JH, Demchak B, Bader GD. Biological network exploration with cytoscape 3. Curr Protoc Bioinformatics. 2014; 47:8.13.1-24. 10.1002/0471250953.bi0813s47 25199793 PMC4174321

