# Supplementary material for: Transcriptome analysis of common and diverged circulating miRNAs between arterial and venous during aging
Source: Aging (Albany NY). 2020 Jun 30;12(13):12987–3004. doi: 10.18632/aging.103385 (PMC7377886; doi:10.18632/aging.103385)
Supplement: Supplementary Table 1 [file aging-12-103385-s001..docx]

**Supplementary Table 1. Aging-related deregulated miRNAs in venous plasma.**

| **ID** | **log_2_FC (****Aged_V/Young_V)** | **P value** | **FDR** |
| --- | --- | --- | --- |
| rno-miR-802-3p | 10.8 | 7.40E-03 | 1.45E-02 |
| rno-miR-122-3p | 3.62 | <1.00E-300 | <1.00E-300 |
| rno-miR-138-5p | 3.32 | 1.04E-03 | 2.58E-03 |
| rno-miR-122-5p | 3.05 | <1.00E-300 | <1.00E-300 |
| rno-miR-31a-3p | 3.03 | 3.28E-03 | 7.05E-03 |
| rno-miR-141-3p | 3 | 1.43E-02 | 2.62E-02 |
| rno-miR-802-5p | 2.66 | 7.44E-16 | 3.77E-15 |
| rno-miR-32-5p | 2.52 | 1.98E-02 | 3.47E-02 |
| rno-miR-1247-5p | 2.47 | 7.11E-07 | 2.58E-06 |
| rno-miR-192-3p | 2.38 | 3.71E-29 | 2.88E-28 |
| rno-miR-145-5p | 2.28 | <1.00E-300 | <1.00E-300 |
| rno-miR-200b-3p | 2.19 | 8.18E-11 | 3.51E-10 |
| rno-miR-10a-3p | 2 | 1.35E-02 | 2.50E-02 |
| rno-miR-194-5p | 1.98 | 1.91E-21 | 1.18E-20 |
| rno-miR-6216 | 1.88 | 1.34E-14 | 6.67E-14 |
| rno-miR-30c-2-3p | 1.82 | 3.14E-02 | 5.01E-02 |
| rno-miR-29c-5p | 1.81 | 2.05E-02 | 3.53E-02 |
| rno-miR-31a-5p | 1.77 | 2.10E-06 | 7.42E-06 |
| rno-miR-132-3p | 1.76 | 2.89E-02 | 4.77E-02 |
| rno-miR-205 | 1.75 | 1.60E-14 | 7.84E-14 |
| rno-miR-664-3p | 1.72 | 1.48E-02 | 2.67E-02 |
| rno-miR-29c-3p | 1.7 | 9.91E-24 | 6.75E-23 |
| rno-miR-378b | 1.6 | 2.17E-03 | 5.10E-03 |
| rno-miR-193a-5p | 1.57 | 2.60E-14 | 1.21E-13 |
| rno-miR-375-3p | 1.57 | 3.39E-11 | 1.48E-10 |
| rno-miR-466b-2-3p | 1.43 | 5.55E-32 | 4.85E-31 |
| rno-miR-466b-4-3p | 1.43 | 5.55E-32 | 4.85E-31 |
| rno-miR-29b-3p | 1.42 | 8.56E-86 | 1.14E-84 |
| rno-miR-466c-3p | 1.33 | 1.14E-26 | 8.37E-26 |
| rno-miR-483-5p | 1.33 | 1.65E-02 | 2.95E-02 |
| rno-miR-466b-3p | 1.31 | 3.01E-25 | 2.15E-24 |
| rno-miR-1249 | 1.25 | 2.45E-03 | 5.62E-03 |
| rno-miR-34b-3p | 1.22 | 2.67E-06 | 9.31E-06 |
| rno-miR-192-5p | 1.2 | 8.35E-174 | 1.79E-172 |
| rno-miR-21-5p | 1.12 | 9.67E-13 | 4.29E-12 |
| rno-miR-206-3p | 1.12 | 5.25E-145 | 9.78E-144 |
| rno-miR-30a-5p | 1.09 | 2.55E-03 | 5.78E-03 |
| rno-miR-429 | 1.09 | 3.19E-09 | 1.25E-08 |
| rno-miR-30e-3p | -1.02 | 5.85E-04 | 1.50E-03 |
| rno-miR-19a-3p | -1.02 | 2.13E-37 | 2.05E-36 |
| rno-miR-142-5p | -1.09 | 2.27E-71 | 2.75E-70 |
| rno-miR-142-3p | -1.1 | 4.78E-06 | 1.63E-05 |
| rno-miR-409a-3p | -1.11 | 6.45E-04 | 1.64E-03 |
| rno-miR-17-5p | -1.12 | 1.55E-18 | 9.19E-18 |
| rno-miR-874-3p | -1.13 | 3.07E-03 | 6.64E-03 |
| rno-miR-483-3p | -1.13 | 5.19E-07 | 1.93E-06 |
| rno-miR-361-5p | -1.16 | 1.83E-03 | 4.42E-03 |
| rno-miR-301a-3p | -1.17 | 2.90E-02 | 4.77E-02 |
| rno-miR-361-3p | -1.19 | 7.90E-05 | 2.32E-04 |
| rno-miR-199a-5p | -1.28 | 2.23E-10 | 9.04E-10 |
| rno-miR-423-3p | -1.34 | 3.62E-30 | 2.89E-29 |
| rno-miR-181a-5p | -1.36 | 2.99E-30 | 2.45E-29 |
| rno-miR-450b-3p | -1.43 | 5.16E-06 | 1.74E-05 |
| rno-miR-18a-3p | -1.44 | 6.25E-05 | 1.92E-04 |
| rno-miR-196b-5p | -1.46 | 9.23E-06 | 3.00E-05 |
| rno-miR-615 | -1.48 | 1.14E-02 | 2.14E-02 |
| rno-let-7e-3p | -1.5 | 3.01E-02 | 4.91E-02 |
| rno-miR-15b-3p | -1.52 | 6.94E-76 | 8.81E-75 |
| rno-miR-16-3p | -1.56 | 3.06E-05 | 9.48E-05 |
| rno-miR-369-5p | -1.62 | 1.62E-02 | 2.91E-02 |
| rno-miR-181c-5p | -1.63 | 5.86E-04 | 1.50E-03 |
| rno-miR-872-5p | -1.66 | 1.95E-04 | 5.38E-04 |
| rno-let-7f-2-3p | -1.68 | 1.65E-03 | 4.05E-03 |
| rno-miR-324-5p | -1.74 | 8.56E-06 | 2.81E-05 |
| rno-miR-450a-5p | -1.82 | 1.05E-02 | 1.99E-02 |
| rno-miR-199a-3p | -1.85 | 8.42E-95 | 1.24E-93 |
| rno-miR-210-3p | -1.86 | 8.11E-25 | 5.66E-24 |
| rno-miR-181b-5p | -1.93 | 3.09E-13 | 1.41E-12 |
| rno-miR-195-3p | -1.94 | 5.24E-03 | 1.07E-02 |
| rno-miR-134-5p | -2.08 | 1.29E-04 | 3.68E-04 |
| rno-miR-450b-5p | -2.08 | 3.01E-04 | 8.07E-04 |
| rno-miR-421-5p | -2.12 | 1.24E-02 | 2.31E-02 |
| rno-miR-322-5p | -2.22 | 2.26E-14 | 1.07E-13 |
| rno-miR-542-5p | -2.22 | 4.07E-09 | 1.58E-08 |
| rno-miR-351-5p | -2.29 | 1.02E-53 | 1.14E-52 |
| rno-miR-140-5p | -2.4 | 1.79E-21 | 1.14E-20 |
| rno-miR-503-5p | -2.66 | 2.84E-03 | 6.20E-03 |
| rno-miR-411-3p | -2.71 | 1.99E-02 | 3.48E-02 |
| rno-miR-136-3p | -2.76 | 1.77E-02 | 3.14E-02 |
| rno-miR-140-3p | -2.97 | 0.00E+00 | 0.00E+00 |
| rno-miR-224-5p | -3.08 | 1.04E-18 | 6.29E-18 |
| rno-miR-152-3p | -3.12 | 7.29E-04 | 1.83E-03 |
| rno-miR-542-3p | -3.19 | 1.33E-07 | 5.02E-07 |
| rno-miR-149-5p | -3.21 | 4.34E-90 | 6.06E-89 |
| rno-miR-329-3p | -3.25 | 4.92E-03 | 1.01E-02 |
| rno-miR-18a-5p | -3.39 | 4.54E-02 | 6.89E-02 |
| rno-miR-382-5p | -3.4 | 9.92E-11 | 4.07E-10 |
| rno-miR-322-3p | -3.69 | 3.91E-203 | 9.09E-202 |
| rno-miR-351-3p | -3.92 | 3.44E-23 | 2.23E-22 |
| rno-miR-455-3p | -3.94 | 9.37E-294 | 2.91E-292 |
| rno-miR-455-5p | -4.12 | 1.74E-14 | 8.39E-14 |
| rno-miR-196a-5p | -4.4 | 3.46E-13 | 1.56E-12 |
| rno-miR-503-3p | -4.51 | 1.39E-148 | 2.76E-147 |
| rno-miR-181a-1-3p | -9.57 | 3.13E-02 | 5.01E-02 |
| rno-miR-382-3p | -9.6 | 3.30E-02 | 5.23E-02 |
| rno-miR-496-3p | -9.77 | 3.55E-02 | 5.60E-02 |
| rno-miR-487b-3p | -9.8 | 3.75E-02 | 5.78E-02 |
| rno-miR-376c-3p | -9.97 | 4.26E-02 | 6.53E-02 |
| rno-miR-3586-3p | -10.27 | 3.75E-02 | 5.78E-02 |
| rno-miR-34b-5p | -10.38 | 3.67E-02 | 5.76E-02 |
| rno-miR-33-5p | -10.67 | 3.82E-03 | 8.02E-03 |
| rno-miR-363-3p | -10.8 | 2.23E-02 | 3.79E-02 |
| rno-miR-181b-1-3p | -11.03 | 6.30E-03 | 1.25E-02 |
| rno-miR-675-3p | -11.36 | 3.77E-03 | 7.97E-03 |
| rno-miR-434-3p | -11.45 | 1.05E-04 | 3.03E-04 |

The exact value for P-value (or FDR) smaller than 1.00E-300 could not be calculated, therefore it is shown as <1.00E-300 instead. FC: Fold Change; V: Vein.
