# Supplementary material for: Transcriptome analysis of common and diverged circulating miRNAs between arterial and venous during aging
Source: Aging (Albany NY). 2020 Jun 30;12(13):12987–3004. doi: 10.18632/aging.103385 (PMC7377886; doi:10.18632/aging.103385)
Supplement: Supplementary Tables 2-4 [file aging-12-103385-s002..pdf]

SUPPLEMENTARY TABLES

Supplementary Table 2. Aging-related deregulated miRNAs in arterial plasma.

ID	log_2FC (Aged_A/Young_A)	P value	FDR
rno-miR-136-3p	11.14	1.95E-02	5.00E-02
rno-miR-7a-2-3p	3.24	2.41E-04	1.19E-03
rno-miR-194-5p	3.05	8.54E-29	1.04E-27
rno-miR-141-3p	2.89	9.35E-03	2.75E-02
rno-miR-802-5p	2.43	1.11E-12	9.43E-12
rno-miR-122-3p	2.38	<1.00E-300	<1.00E-300
rno-miR-204-5p	2.37	8.18E-04	3.47E-03
rno-miR-200a-3p	2.16	3.49E-03	1.27E-02
rno-miR-122-5p	2.09	<1.00E-300	<1.00E-300
rno-let-7e-3p	1.88	3.81E-02	8.68E-02
rno-miR-483-3p	1.77	1.74E-02	4.62E-02
rno-miR-192-3p	1.16	4.30E-16	3.86E-15
rno-miR-29b-3p	1.15	2.53E-24	2.86E-23
rno-miR-192-5p	1.13	3.15E-146	1.37E-144
rno-miR-26b-3p	1.12	4.26E-03	1.49E-02
rno-miR-199a-5p	-1	2.89E-05	1.58E-04
rno-miR-193b-5p	-1.01	2.94E-02	7.07E-02
rno-miR-27a-5p	-1.03	2.06E-02	5.24E-02
rno-miR-324-5p	-1.05	1.26E-02	3.47E-02
rno-miR-149-5p	-1.08	4.73E-14	4.12E-13
rno-miR-199a-3p	-1.17	6.25E-23	6.82E-22
rno-miR-224-5p	-1.19	1.27E-04	6.56E-04
rno-miR-107-3p	-1.19	8.67E-03	2.62E-02
rno-miR-497-5p	-1.24	3.66E-03	1.31E-02
rno-miR-382-5p	-1.25	8.94E-04	3.74E-03
rno-miR-181a-5p	-1.31	6.56E-23	6.91E-22
rno-miR-99b-3p	-1.31	4.77E-03	1.62E-02
rno-let-7d-5p	-1.33	1.94E-39	2.69E-38
rno-miR-3074	-1.42	4.78E-02	1.03E-01
rno-let-7i-3p	-1.45	3.88E-02	8.77E-02
rno-miR-142-3p	-1.45	1.75E-06	1.05E-05
rno-let-7i-5p	-1.48	5.79E-43	8.84E-42
rno-miR-181b-5p	-1.51	1.18E-08	9.24E-08
rno-let-7c-1-3p	-1.53	1.17E-02	3.37E-02
rno-miR-140-5p	-1.54	6.72E-09	5.40E-08
rno-miR-542-5p	-1.57	2.07E-08	1.58E-07
rno-miR-342-5p	-1.6	9.86E-03	2.87E-02
rno-miR-450b-5p	-1.6	1.83E-02	4.76E-02
rno-miR-17-5p	-1.63	2.87E-42	4.17E-41
rno-miR-181c-5p	-1.65	6.87E-03	2.16E-02
rno-miR-7a-5p	-1.72	6.38E-03	2.05E-02
rno-miR-455-3p	-1.75	1.69E-85	6.46E-84
rno-miR-140-3p	-1.79	<1.00E-300	<1.00E-300

rno-miR-322-5p	-1.8	2.36E-07	1.55E-06
rno-miR-434-3p	-1.83	3.05E-02	7.22E-02
rno-miR-195-3p	-1.86	5.94E-03	1.96E-02
rno-miR-667-3p	-1.86	2.71E-02	6.61E-02
rno-miR-450a-5p	-1.91	4.98E-04	2.24E-03
rno-miR-322-3p	-1.98	6.83E-49	1.16E-47
rno-miR-10b-3p	-1.98	2.70E-02	6.61E-02
rno-miR-652-3p	-1.99	1.72E-02	4.59E-02
rno-miR-181d-5p	-2.13	3.73E-02	8.55E-02
rno-miR-503-5p	-2.27	1.77E-03	6.93E-03
rno-miR-296-5p	-2.31	1.68E-02	4.53E-02
rno-miR-144-3p	-2.36	1.99E-03	7.69E-03
rno-miR-20b-5p	-2.38	7.56E-04	3.25E-03
rno-miR-133b-3p	-2.47	1.51E-03	5.98E-03
rno-miR-466c-5p	-2.57	1.88E-07	1.30E-06
rno-miR-152-3p	-2.68	3.30E-06	1.94E-05
rno-miR-455-5p	-2.86	7.70E-12	6.35E-11
rno-miR-345-5p	-3.29	2.07E-03	7.90E-03
rno-miR-106b-5p	-3.43	3.51E-03	1.27E-02

The exact value for P-value (or FDR) smaller than 1.00E-300 could not be calculated, therefore it is shown as <1.00E-300 instead. FC: Fold Change; A: Artery.

Supplementary Table 3. Arterial-versus-venous differentially-expressed miRNAs in the young group.

ID	log_2FC (Young_A/Young_V)	P value	FDR
rno-miR-6324	4.03	4.95E-03	1.48E-02
rno-miR-138-5p	3.9	3.64E-04	1.47E-03
rno-miR-935	3.5	3.08E-02	7.48E-02
rno-miR-125b-2-3p	3.27	3.32E-02	7.91E-02
rno-miR-132-3p	2.76	1.70E-04	7.18E-04
rno-miR-450a-5p	2.25	2.76E-06	1.54E-05
rno-miR-100-5p	2.19	8.61E-03	2.40E-02
rno-miR-200b-3p	2.03	1.21E-05	6.18E-05
rno-miR-466b-5p	1.94	3.10E-02	7.48E-02
rno-miR-29c-5p	1.89	2.45E-02	6.14E-02
rno-let-7i-3p	1.77	3.11E-02	7.48E-02
rno-miR-145-5p	1.68	<1.00E-300	<1.00E-300
rno-miR-20a-5p	1.67	1.41E-02	3.75E-02
rno-miR-28-5p	1.66	5.44E-05	2.55E-04
rno-miR-193a-5p	1.65	5.78E-09	4.07E-08
rno-miR-466c-5p	1.51	1.14E-05	5.89E-05
rno-miR-378b	1.45	1.14E-02	3.12E-02
rno-miR-205	1.4	1.38E-05	6.89E-05
rno-miR-31a-5p	1.39	7.65E-03	2.18E-02
rno-miR-34a-5p	1.36	3.80E-02	8.78E-02
rno-miR-122-3p	1.28	1.63E-35	3.02E-34
rno-miR-29c-3p	1.28	1.26E-08	8.67E-08
rno-miR-26a-5p	1.26	2.25E-03	7.48E-03
rno-miR-222-3p	1.24	9.50E-04	3.47E-03
rno-miR-125b-5p	1.21	1.95E-47	5.76E-46
rno-miR-152-3p	1.2	3.40E-04	1.40E-03
rno-miR-142-3p	1.18	2.31E-05	1.12E-04
rno-miR-99a-5p	1.17	3.63E-02	8.52E-02
rno-miR-143-3p	1.15	2.80E-47	7.53E-46
rno-miR-192-3p	1.13	3.59E-03	1.10E-02
rno-miR-1249	1.11	4.44E-03	1.34E-02
rno-miR-375-3p	1.11	1.38E-03	4.87E-03
rno-miR-450b-5p	1.08	1.69E-02	4.47E-02
rno-miR-148a-3p	1.06	8.24E-03	2.32E-02
rno-miR-30a-5p	1.02	2.79E-02	6.92E-02
rno-miR-221-3p	1.01	2.27E-07	1.40E-06
rno-miR-206-3p	1	4.24E-05	2.02E-04
rno-miR-15b-3p	-1.06	4.02E-29	5.66E-28
rno-miR-26b-3p	-1.07	3.78E-04	1.51E-03
rno-miR-30e-3p	-1.09	2.53E-25	2.87E-24
rno-miR-503-3p	-1.16	5.94E-22	6.51E-21

rno-miR-511-5p	-1.18	4.09E-02	9.22E-02
rno-miR-223-5p	-1.27	1.01E-04	4.49E-04
rno-miR-196a-5p	-1.39	3.16E-03	1.01E-02
rno-miR-200a-3p	-1.59	5.59E-03	1.65E-02
rno-miR-3559-5p	-1.73	1.00E-02	2.77E-02
rno-miR-191a-3p	-1.89	1.93E-07	1.21E-06
rno-miR-16-3p	-1.89	1.44E-04	6.26E-04
rno-miR-7a-2-3p	-1.9	4.25E-02	9.53E-02
rno-miR-483-3p	-2.1	3.98E-04	1.57E-03
rno-miR-136-3p	-10.61	4.05E-02	9.21E-02
rno-miR-33-5p	-10.67	3.61E-02	8.52E-02
rno-miR-323-3p	-11.47	4.57E-02	1.01E-01
rno-miR-421-5p	-11.84	1.56E-03	5.35E-03

The exact value for P-value (or FDR) smaller than 1.00E-300 could not be calculated, therefore is shown as <1.00E-300 instead. FC: Fold Change; A: Artery; V: Vein.

Supplementary Table 4. Arterial-versus-venous differentially-expressed miRNAs in the aged group.

ID	log_2FC (Aged_A/Aged_V)	P value	FDR
rno-miR-675-3p	11.18	1.91E-03	1.25E-02
rno-miR-18a-5p	3.82	2.33E-02	9.90E-02
rno-miR-136-3p	3.29	5.03E-03	2.80E-02
rno-miR-196a-5p	3.18	9.97E-04	7.61E-03
rno-miR-503-3p	2.74	4.96E-31	1.59E-29
rno-miR-143-5p	2.63	1.90E-02	8.39E-02
rno-miR-351-3p	2.55	8.22E-08	1.12E-06
rno-let-7e-3p	2.54	2.24E-04	2.02E-03
rno-miR-542-3p	2.52	2.20E-03	1.38E-02
rno-miR-500-3p	2.38	4.77E-03	2.72E-02
rno-miR-26a-3p	2.23	3.59E-02	1.33E-01
rno-miR-450a-5p	2.16	8.86E-04	7.00E-03
rno-miR-455-3p	2.13	2.46E-54	1.11E-52
rno-miR-369-5p	2.04	2.79E-02	1.11E-01
rno-miR-351-5p	1.98	2.93E-36	1.10E-34
rno-miR-224-5p	1.78	1.58E-03	1.09E-02
rno-miR-149-5p	1.76	2.70E-21	6.77E-20
rno-miR-450b-5p	1.55	1.69E-03	1.14E-02
rno-miR-542-5p	1.46	2.16E-03	1.37E-02
rno-miR-140-5p	1.41	1.43E-08	2.30E-07
rno-miR-322-3p	1.36	6.51E-15	1.22E-13
rno-miR-18a-3p	1.36	4.23E-03	2.48E-02
rno-miR-450b-3p	1.33	2.72E-05	2.92E-04
rno-miR-196b-5p	1.29	1.79E-02	8.04E-02
rno-miR-331-3p	1.25	1.10E-03	8.14E-03
rno-miR-23b-5p	1.23	2.67E-02	1.07E-01
rno-miR-140-3p	1.22	1.15E-88	1.30E-86
rno-miR-872-5p	1.19	2.63E-02	1.07E-01
rno-miR-615	1.15	3.01E-02	1.18E-01
rno-miR-106b-3p	1.01	1.21E-02	5.91E-02
rno-miR-466c-5p	-1.01	1.05E-02	5.29E-02
rno-miR-223-5p	-1.09	9.86E-03	5.05E-02
rno-miR-187-3p	-1.1	6.65E-03	3.61E-02
rno-miR-378b	-1.12	1.82E-02	8.10E-02
rno-miR-7a-5p	-1.13	8.39E-03	4.45E-02
rno-miR-652-3p	-1.24	4.86E-02	1.71E-01
rno-miR-144-3p	-1.32	3.25E-02	1.23E-01
rno-miR-133b-3p	-1.69	8.60E-03	4.50E-02
rno-miR-1949	-1.74	3.05E-02	1.18E-01
rno-miR-328a-5p	-2.77	2.81E-02	1.11E-01
rno-miR-31a-3p	-4.28	4.51E-03	2.61E-02
rno-miR-188-5p	-10.8	9.19E-03	4.76E-02

FC: Fold Change; A: Artery; V: Vein.
